# *In Vitro* and *In Vivo* Survival and Colonic Adhesion of *Pediococcus acidilactici* MTCC5101 in Human Gut

**DOI:** 10.1155/2013/583850

**Published:** 2013-09-24

**Authors:** Praveen P. Balgir, Baljinder Kaur, Tejinder Kaur, Natisha Daroch, Gurpreet Kaur

**Affiliations:** Department of Biotechnology, Punjabi University, Punjab, Patiala 147 002, India

## Abstract

The present study aims to investigate the probiotic nature of *Pediococcus acidilactici* MTCC5101 by an *in vitro* assay of bacterial adherence to intestinal epithelial cells of human gastrointestinal (GI) tract using Caco-2 cell line. Further to assess the *in vivo* survival in the GI tract, oral feeding was carried out with the help of 10 healthy volunteers. The effect on wellness was assessed by studying blood biochemical parameters of the volunteers. The survival of the bacteria was assessed using PCR-based detection of *P. acidilactici* MTCC5101 in fecal samples. The probiotic nature of *P. acidilactici* MTCC 5101 was strengthened by its adherence to the intestinal epithelial Caco-2 cell line in the *in vitro* SEM observations. Oral feeding study for assessing the survival of bacteria in GI tract of volunteers showed the strain to be established in the GI tract which survived for about 2 weeks after feeding.

## 1. Introduction

Human intestinal microbiota is a metabolically active microbial environment that is relatively stable in the guts of healthy individuals [1]. The commensal intestinal flora of humans and animals includes the genera *Lactobacillus*, *Pediococcus,* and *Lactococcus* [2, 3]. Strains of these genera are frequently used on large scale as starter cultures in food industries because of their generally recognized as safe (GRAS) and probiotic status [4–6]. 

Probiotics are known to exert protective influence in the human intestine through various mechanisms and represent promising applications in prophylaxis and therapy. It has also been acknowledged by WHO that probiotic microorganisms when administered in adequate amounts confer a health benefit to the host [7]. Improvement of health status by probiotics can be explained by the fact that these bacteria reduce production of toxic substances, aid in production and absorption of vital nutrients, stimulate gastrointestinal activity as well as immunity, and reduce colonization of anaerobic pathogens by competitive exclusion [8, 9]. 

For all the health giving effects, the probiotic bacteria should also be capable of surviving while passage through the GI tract. It is essential for them to overcome highly acidic environment of stomach, digestive enzymes, and bile salts of the small intestine, which thus become important selection criterion for probiotic strains. Apart from this, adherence of bacterial cells to intestinal epithelial cells and/or mucus is considered to be a desirable feature of probiotics, as it promotes enhanced gut residence time, pathogen exclusion, and interaction with host epithelial and immune cells [10, 11]. 

*Pediococcus acidilactici* MTCC5101 is an acid and bile tolerant probiotic strain that secretes a potent antibacterial bacteriocin designated as Pediocin CP2. Pediocin CP2 exhibits a wide range of antimicrobial activity against Gram-positive, Gram-negative bacteria as well as fungi [12]. Genes encoding production of Pediocin CP2 are localized to the *ped *operon present on the 8.9 kb plasmid pCP289 of *P. acidilactici* MTCC5101 [13–15]. These properties make this sp. of *Pediococcus* an attractive prophylactic and therapeutic agent against pathogenic bacteria in GI tract. However, the final confirmation of its probiotic nature can come only from human trials. It is these trials that provide evidence of the survival of the probiotic strain *in vivo* and provide the required basis for a credible claim.

The present study aims to investigate the probiotic nature of *P. acidilactici *MTCC5101 by an *in vitro* assay of bacterial adhesion to intestinal epithelial cells of human GI tract using Caco-2 cell line. Further, to assess the *in vivo* survival in the GI tract, oral feeding was carried out with the help of 10 healthy volunteers. The effect on wellness was assessed by studying blood biochemical parameters of the volunteers. 

## 2. Materials and Methods

### 2.1. Bacterial Strain Procurement and Maintenance

*Pediococcus acidilactici* MTCC5101, characterized in the laboratory of Department of Biotechnology, Punjabi University, Patiala [12], was used in the present study. It was revived and maintained at 37°C for 18–24 h under microaerophilic conditions in de Man, Rogosa, and Sharpe (MRS) broth [16].

### 2.2. *In Vitro* Adhesion Assay of *Pediococcus acidilactici* MTCC5101

The enterocyte-like Caco-2 cells were obtained from National Centre for Cell Science (NCCS), Pune. Cells were routinely grown in Dulbecco modified Eagle's minimal essential medium containing 25 mM glucose, 10% fetal bovine serum, and 1% antibiotic solution containing penicillin and streptomycin (Himedia) [15]. 

The adherence of pediococci to Caco-2 cells was examined as described previously by Bernet et al. [17] with a few modifications. For the adhesion assay, monolayers of Caco-2 cells were prepared on glass coverslips placed in six-well tissue culture plates seeded at a concentration of 10^6^ cells/mL. Experiments were carried out at 37°C in a 5% CO_2_ atmosphere. The culture medium was changed every alternate day and monolayers at late postconfluence stage (after 15 days) were used for adhesion assay. To begin with the assay, monolayers were washed twice with phosphate-buffered saline (PBS, pH 7.2). 1 mL bacterial culture containing one million CFU/mL suspended in PBS was added to the monolayers on glass coverslips placed in tissue culture plates containing DMEM without antibiotic solution. The plates were incubated for 1 h in 5% CO_2_ at 37°C. After 1 h, the monolayers were washed five times with sterile PBS and fixed for SEM observations. The adhesion assay was repeated thrice.

### 2.3. Scanning Electron Microscopy (SEM)

For SEM analysis, Caco-2 cells cocultured with bacterial cells were fixed for 1 h with 2.5% glutaraldehyde in 0.1 M phosphate buffer (PB, pH 7.4) at room temperature. The cells were washed twice with PB and postfixed for 30 min with 2% osmium tetroxide in PB. After fixation, cells were again washed thrice with PB and then dehydrated in a graded series (30, 50, 70, 80, 90, and 100%) of ethanol [17]. Instead of critical point dryer, cells were treated with 100% hexamethyldisilazane for 10 min and coated with gold [18]. The specimens were examined with a JEOL JSM-6610LV scanning electron microscope at Indian Institute of Technology, Ropar, Punjab, India. A number of bacterial cells adhered onto Caco-2 cells were counted on various microscopic fields per coverslip while performing SEM. The values were represented as average number of bacterial cells adhered per 100 Caco-2 cells.

### 2.4. *In Vivo* Study Design

The study was approved by the Institutional Clinical Ethics Committee vide clearance no. ICEC/3/2011. Experiments were strictly performed according to the guidelines of Indian Council of Medical Research for conducting research on human trials. Ten healthy volunteers were selected after taking their informed consent. It was a controlled study consisting of parallel 6 weeks trials, with 4 weeks of intervention and 2 weeks wash-out period. 

#### 2.4.1. Preparation of Probiotic Buttermilk

The overnight grown *P. acidilactici* MTCC5101 cells were harvested by centrifugation (6000 rpm, 10 min) and resuspended slowly in PBS (pH 8.0). Cells at a concentration of 10^8^–10^10^ cells/mL were added to fresh buttermilk as medium for oral feeding study (Verka Co. Ltd., Patiala, India). Cell counts were taken on a haemocytometer [19] and after thorough microbiological examination, cells at an average dosage level of 10^8^–10^10^ cells/mL/day were fed to 10 healthy subjects in 1 mL buttermilk base, administered once a day for 4 weeks continuously [20]. Equivalent amount of buttermilk without *P. acidilactici* MTCC5101 was fed to a group of 3 control volunteers which served as negative controls. The subjects were asked to abstain from taking other fermented milk products and supplements with probiotics during the trial period. They were explained how to collect, store, and deliver the fecal samples. Participants were asked about changes in their wellbeing and/or had taken any kind of medication during study period.

#### 2.4.2. Collection and Analysis of Fecal Samples

The fecal swab samples were collected using ear buds in 2 mL of sterile normal saline (0.85% NaCl) after feeding trials of 5, 10, 20, and 30 days [38]. The fecal suspension was cultured in MRS broth for bacterial enumeration for 18 h at 37°C. Cell pellets were harvested by centrifugation and were stored at −20°C for plasmid DNA isolation. 

#### 2.4.3. Molecular Identification of *P. acidilactici* MTCC5101 in Fecal Samples

A metagenomic approach was followed for the isolation of plasmid DNA from fecal samples [39]. Isolated DNA was stored at −20°C until further use and its quality, quantity, and purity were determined using agarose gel electrophoresis and absorbance measurements on spectrophotometer (UV mini-1240, UV-Vis spectrophotometer, Shimadzu Corporation). PCR was performed in PROGENE (Techne) using forward primer 5′-CTGCGTTGATAGGCCAGGT-3′ and reverse primer 5′-ACCTTGATGTCCACCAGTAGC-3′ which are specific for 323 bp fragment of *pedA* gene located on the cryptic plasmid pCP289 of *P. acidilactici* MTCC5101 [13]. The amplification program consisted of 1 cycle of 94°C for 3 min and 30 cycles of amplification (94°C for 1 min, 63°C for 1 min, and 72°C for 1 min).

#### 2.4.4. Bacteriocin Activity Assay

Well diffusion assay was performed as per standard methodology of Sarkar and Banerjee [40]. MRS hard agar (1% w/v) was overlayed with MRS soft agar (0.75% w/v) preseeded with approximately one million cells of indicator *Enterococcus faecalis*. Wells were filled with heat inactivated supernatant of MRS grown fecal samples pertaining to the 30th day of intervention and plates were incubated at 37°C for 24 h.

### 2.5. Determination of Wellness Parameters

To check the effect of the probiotic on the wellness of test subjects, blood samples were drawn 2 weeks before and 2 weeks after the intervention. Wellness parameters such as total white blood cells (WBC) and red blood cells (RBC) counts, Hb (Haemoglobin), bleeding, and clotting time were recorded.

### 2.6. Statistics

Statistical analysis of data was performed using the Daniel's XL Toolbox, version 5.09. For the cell counts and hematological analysis, significant differences in the observed values were tested using one-way analysis of variance (ANOVA). When significant differences were found, multiple comparison/post hoc testing based on Bonferroni-Holm was carried out. Differences were considered statistically significant when *P* < 0.05.

## 3. Results

### 3.1. Adhesion of *P. acidilactici* MTCC5101 to Caco-2 Cells

**Figure 1:**
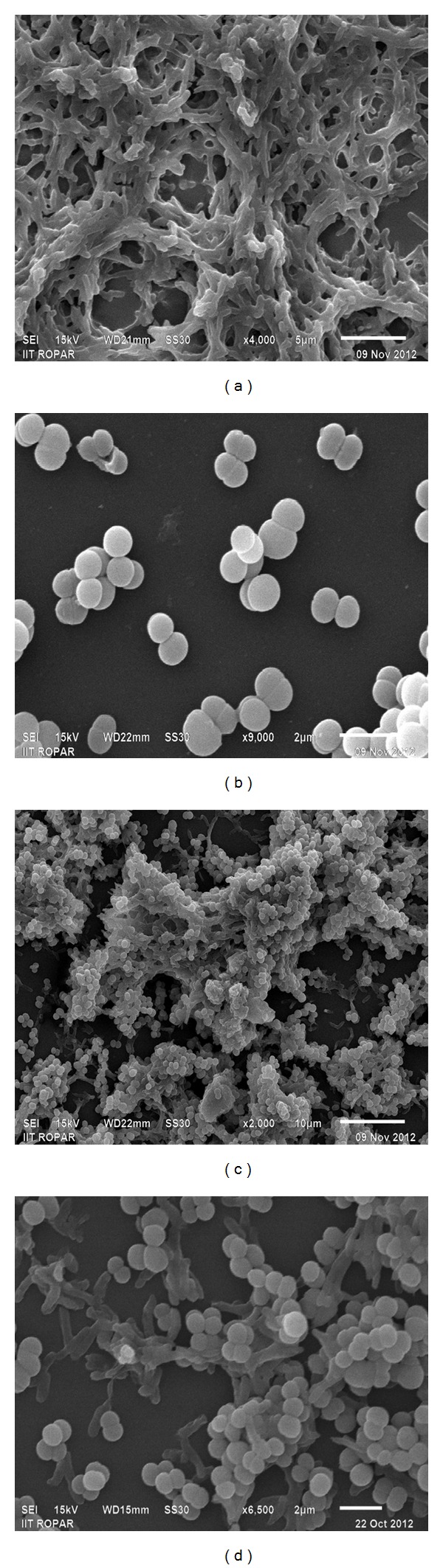
SEM micrographs of (a) untreated Caco-2 cells (magnification level 4,000x) (b) *P. acidilactici *MTCC 5101 (magnification level 9,000x), and (c) and (d) adherent *P. acidilactici *MTCC 5101 on Caco-2 cells (magnification level 2,000x and 6,500x).

Figures 1(a) and 1(b) represent SEM micrographs of untreated Caco-2 cells and *P. acidilactici* MTCC5101 with their typical tetrad arrangement. After examining the SEM images of cocultured *P. acidilactici *MTCC5101 with Caco-2 cells, it is clearly illustrated that the selected probiotic has a very high tendency to adhere to intestinal epithelium or Caco-2 cells at the mucosal surface. Adhesion of selected probiotic strain onto monolayers of Caco-2 cells was evaluated on different microscopic fields per coverslip and at an average 152 ± 33 cells adhered per 100 Caco-2 cells. A biofilm of adherent bacteria constituted of pediococci was observed. The bacteria were also found to adhere to each other (Figures 1(c) and 1(d)).

### 3.2. Total LAB Count in Fecal Samples

In Table 1 the total cell counts of *P. acidilactici* MTCC5101 in buttermilk base are recorded. All the subjects have received oral doses of 10^8^–10^10^ cells/mL/day of *P. acidilactici* in buttermilk base. The fecal swab samples cultured in MRS medium showed growth of mixed LAB with cell counts of 9.27 ± 1.01 cells/mL at the baseline (day 0) and 9.31 ± 0.98 cells/mL after 4 weeks of intervention. Similarly, fecal swab samples from control group showed cell counts of 10.17 ± 1.06 cells/mL at the baseline and 9.76 ± 0.33 cells/mL after 4 weeks (Table 2). 

#### 3.2.1. Molecular Identification of *P. acidilactici* MTCC5101 in Fecal Samples

**Figure 2:**
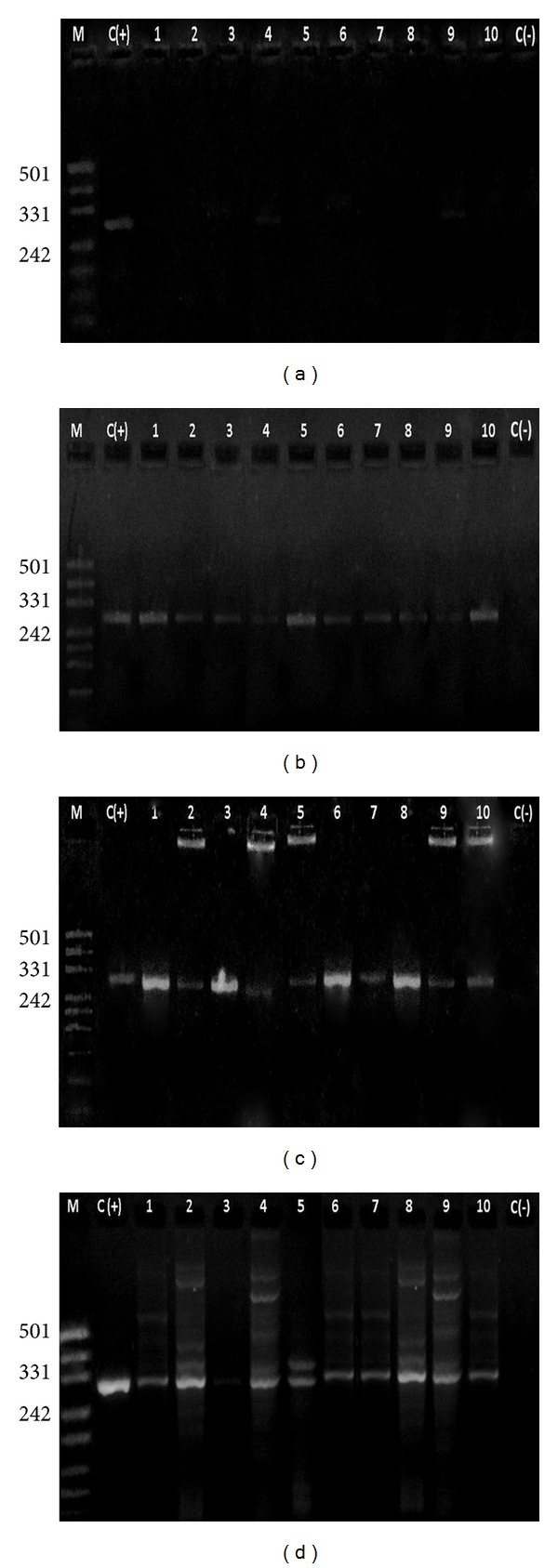
2% agarose gel showing 323 bp DNA fragment amplified using plasmids from fecal samples (a) M: marker pUC 19/Msp Digest, C(+): positive control, Lane 1–10: 5th day samples, C(−): negative control. (b) M: marker pUC 19/Msp digest, C(+): positive control, lane 1–10: 10th day's samples, C(−): Negative control. (c) M: Marker pUC 19/Msp Digest, C(+): Positive control, Lane 1–10: 20th day's samples, C(−): negative control. (d) M: marker pUC 19/Msp Digest, C(+): positive control, lane 1–10: 30th day's samples, C(−): negative control.

Results of the PCR analysis coincide with count data that indicates detectable levels of *P. acidilactici *MTCC5101 in all the volunteers after a 4-week intervention. Figures 2(a) and 2(b) depict agarose gel analysis of 323 bp fragment of partial *pedA *gene amplified from isolated whole LAB plasmids of 5th and 10th day's fecal samples. Intensity of the PCR amplicon is lesser in the 5th day's samples which show that the establishment of the probiotic strain requires a little longer time to persist and colonize in the human gut. The fragment of the expected size was obtained in nearly all 10th day's samples and the bands in agarose gels were more prominent as compared to 5th day which confirms that the pediococci have started gut colonization. A similar pattern is observed in the 20th and 30th day's fecal samples on 2% agarose (Figures 2(c) and 2(d)). The band intensity was more prominent as compared to 5th and 10th day samples which indicate that the strain has colonized in the GI tract of volunteers, hence proving that colonization progresses in a time-dependent manner. 

#### 3.2.2. Bacteriocin Activity Assay

**Figure 3:**
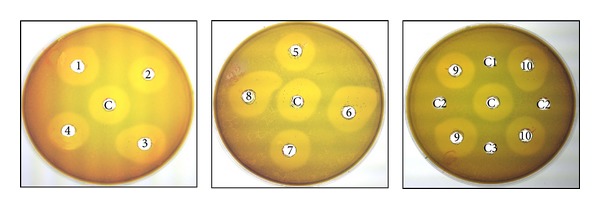
Well diffusion assay of cultured fecal samples of 30th day's against *E. faecalis*. C: *P. acidilactici *MTCC 5101; C1–C3: Fecal swab samples of volunteer controls; 1–10: Fecal swab samples of volunteer subjects.

To check for pediocin CP2 profile in the mixed fecal cell cultures of 30th day, standard well diffusion assay was carried out using *E*. *faecalis* as the indicator strain. After 24 h, definite zone of inhibition was seen in the plates that also confirms our previous findings on successful establishment of *P. acidilactici* MTCC5101 in GI tract of volunteers (Figure 3). 

### 3.3. Wellness Parameters Performed

Routine blood tests were performed to estimate the effect of probiotics on wellness parameters of volunteers. The tests include WBC and RBC counts, levels of Hb in blood, bleeding time, and clotting time (Table 3). Haematological survey was carried out before as well as after feeding trial to estimate the effect of probiotics on some of the wellness parameters of test subjects. Results indicated a small yet insignificant increase in the values of RBC counts and Hb levels of subjects. The findings confirm the safe oral consumption and health improvement capacity of probiotic strain.

## 4. Discussion

Bacterial adhesion to epithelial cells in gut is initially based on nonspecific physical interactions between the two surfaces [41, 42]. After primary attachment to epithelial surface, secondary interactions between bacterial adhesins and complementary epithelial receptors play a key role in adhesion of bacterial cells to intestinal mucin and enterocytes [22, 43–47]. Since there is difficulty in studying bacterial adhesion *in vivo*, intestinal cell lines are widely used as *in vitro* models for assessment [48]. We have used a well-characterized cultured colon carcinoma Caco-2 cell line displaying typical features of enterocytic differentiation in the form of villi to study adhesion of *P. acidilactici *MTCC5101. A strong adherence of probiotic strains to intestinal epithelial cells has been reported previously in a number of studies (Table 4). In a recent study by Jensen et al. [49], it has been reported that the adhesion capacity of probiotics varies from species to species as a variation from 1% to 25% has been observed in case of 18 known probiotic lactobacilli and pediococci. The current study provides clear evidence that *P. acidilactici *MTCC5101 adheres strongly to villi of Caco-2 cells. These results further strengthened the claim of this strain for selection as a probiotics for human use.

Oral consumption of probiotics has been advocated with prophylactic and curative properties that have been observed in case of intestinal disorders such as antibiotic-induced diarrheal disease, viral and bacterial diarrhea, lactose intolerance, and inflammatory bowel diseases [27, 29, 34, 50–55]. Recently, there has been accumulation of evidence from rigorous clinical studies on well-characterized probiotics having real health-promoting properties [56, 57]. 

Although minimum effective dose is not known exactly, usually an oral dose of 10^6^ CFU/day or more than this has been followed in most studies (Table 5). Previous clinical and colonization studies prove successful intestinal colonization of probiotic bacteria and prevention of diseased condition. A comparative analysis of such *in vivo *clinical studies demonstrates that the persistence time of probiotic bacteria in gut varies from strain to strain (Table 5). The relative strain-specific persistence *in vivo* correlates accurately and significantly with *in vitro* outcomes as evident from a recent study on *Lactobacillus plantarum *[58]. Studies on probiotic *Lactobacillus casei* strain DN-114 001 and *L. casei* strain Shirota have proven the capacity of these strains to survive and colonize human gut [59, 60]. Persistence of probiotic strains in GI tract is also demonstrated by their bile and acid resistance properties, as shown in earlier study carried out on the present strain [12, 61–64].

Survival in the GI tract depends on both the strain and the food matrix involved [65]. Fecal recovery of several probiotic strains has been demonstrated in different food matrices, including fermented milk and yoghurt [66, 67], fruit drinks [68], supplements [36, 69], and infant formula [65]. The survival of probiotics in human GI tract should lead to shedding of live cells in fecal samples which can be detected using quantitative methods like PCR [37, 70]. In the present parallel, controlled human intervention study, *in vivo* persistence and colonization of *P. acidilactici *MTCC5101 in GI tract provides a clear evidence for intimate interactions between the selected probiotic bacteria and intestinal mucosal surface. These interactions allow probiotic strain to persist in gut for a considerable time period, regardless of the dietary and physiological differences among individuals selected in the study. Furthermore, results indicate that buttermilk is a suitable carrier medium for *P. acidilactici *MTCC5101 strengthening the use of buttermilk as a probiotic product.

Abnormal blood biochemical parameters are an indicator of a number of clinical disorders. Oral consumption of probiotics has not been linked to any adverse subclinical effects on blood biochemistry so far [71, this study]. Probiotics have been reported to enhance absorption of essential vitamins and minerals from the diet into the body [72, 73]. The enhanced absorption of vitamins and minerals has led to improved haematological environment and gut health. A slight increase in RBC counts and Hb levels of volunteer subjects after oral feeding with probiotics strain was observed. Both *in vitro* models and *in vivo* studies have suggested the successful establishment of *P. acidilactici *MTCC5101 in human gut that is being proposed herein with the possibility of providing beneficial health effects to the host.

## 5. Conclusions

In conclusion, *P. acidilactici *MTCC5101 can survive passage through the human GI tract when administered orally in a buttermilk food base. Overall, results indicate that *P. acidilactici *MTCC5101 is a safe and potent probiotic strain with strong adhesive and health-improving characteristics. The findings suggest an opportunity for successful use of *P. acidilactici *MTCC5101 in functional food applications in future.

## Figures and Tables

**Table 1:** Viable cell counts of *P. acidilactici* MTCC5101 in buttermilk base.

Food base	Bacterial count^a^ (cells/mL)			
	Week 1	Week 2	Week 3	Week 4
*P. acidilactici* MTCC5101/mL of buttermilk base	9.9 × 10^8^	1.01 × 10^9^	1 × 10^10^	1.02 × 10^10^

^a^Values are expressed as cells/mL.

**Table 2:** Total LAB counts enumerated in fecal samples.

Interval (days)	Bacterial counts (cells/mL)^a^	
	VC (*n* = 3)	VS (*n* = 10)
At baseline (day 0)	10.17 ± 1.06	9.27 ± 1.01
After intervention (day 30)	9.76 ± 0.33	9.31 ± 0.98

^a^Values are expressed as mean ± SD of log^10^ values.

VC: volunteer controls.

VS: volunteer subjects.

(*n*): no. of individuals.

**Table 3:** Haematological analysis of healthy human subjects.

Haematological tests	Interval	Response in volunteers^a^	
		VC (*n* = 3)	VS (*n* = 10)
WBC counts (per mm^3^ of blood)	Before	3.73 ± 0.02	3.71 ± 0.05
	After	3.71 ± 0.03	3.70 ± 0.04
RBC counts (per mm^3^ of blood)	Before	6.63 ± 0.02	6.56 ± 0.06
	After	6.62 ± 0.02	6.66 ± 0.03
Hb (gm/dL)	Before	0.98 ± 0.02	0.95 ± 0.03
	After	0.98 ± 0.05	3.15 ± 4.4
Bleeding time (sec)	Before	2.09 ± 0.03	2.04 ± 0.11
	After	2.10 ± 0.03	2.04 ± 0.13
Clotting time (sec)	Before	2.35 ± 0.08	2.34 ± 0.07
	After	2.34 ± 0.07	2.35 ± 0.06

^a^Values are expressed as mean ± SD of log^10^ values.

VC: volunteer controls.

VS: volunteer subjects.

(*n*): no. of individuals.

**Table 4:** Adhesion studies based on Probiotics.

Organism(s)	Cells/cell line(s)	Adhesion index*	Reference
*Bifidobacterium breve* (4, 5, 25) *B. longum* (4, 16, 18, 22) *B. bifidum* (7, 8) *B. infantis *(1)	Caco-2 HT29-MTX	*B. breve* (4): 205 ± 18 *B. longum* (16): 72 ± 13 *B. bifidum* (8): 35 ± 6 *B. infantis* (1): 161 ± 14	[17]

*Lactobacillus rhamnosus* GG *L. casei* strain Shirota *L. johnsonii* La1 *L. rhamnosus* LC 705 *B. lactis* Bb12	Mucus from feces	*L. rhamnosus* GG: 28.8% *L. johnsonii* La1: 27.3% *B. lactis* Bb12: 10.0% *L. casei* strain Shirota: 4.0% *L. rhamnosus* Lc705: 1.3%	[21]

*L. rhamnosus* GG *B. animalis* subsp. *lactis* Bb12 *B. animalis* IATA-A2 *B. bifidum* IATA-ES2	Caco-2 HT29-MTX	HT29-MTX: 0.5–2.3% Caco-2/HT29-MTX: 0.6–3.2%	[22]

*L. plantarum *(9, 72, 75, 77, 90, 91) *L. delbrueckii* subsp. *bulgaricus* CH4 *L. plantarum* CSCC5276	HT-29 Caco-2	*L. plantarum* Lp91: 12.8 ± 1.56	[23]

163 *Lactobacillaceae* sp.	HT-29 HT-29 MTX	*HT-29* Pediococci: 12.51% ± 1.4% Lactobacilli: 4.8% ± 61.6% *HT-29 MTX* Pediococci: 13.5% ± 62.0% Lactobacilli: 10.3% ± 62.4%	[24]

*P. acidilactici *MTCC 5101	Caco-2	*P. acidilactici*: 152 ± 33	Present study

*Adhesion is indexed as % adhesion or mean ± SD of the number of bacterial cells adhered per 100 cells of cell line used.

**Table 5:** Studies of oral dosage levels of probiotics in healthy volunteers and patients.

Organism(s)	Subjects	Dose levels (CFU/mL/day)	Response/outcomes	Reference
*Lactobacillus paracasei* subsp. *paracasei* (CRL-431) *L. acidophilus *	Children and adults with diarrhea	10^7^-10^8^	Three 15 day trial periods; reduction in incidence of intestinal disorders	[25]
*Lactobacillus* GG	Healthy volunteers	10^8^–10^10^	1-week trial; effective gut colonization	[26]
*Bifidobacterium lactis* BB-12 *Lactobacillus *GG	Children with atopic eczema	10^8^-10^9^	2-month trial; controlled allergic reactions	[27]
*B. longum* SBT2928	Healthy volunteers	10^11^	40-day trial; survival in the gut	[28]
*Lactobacillus* GG	Children with acute infectious diarrhea	10^9^	Prophylactic; reduction in duration of diarrhea	[29]
*L. casei *subsp. *rhamnosus *Lcr35	Healthy volunteers	10^8^–10^12^	1-week study; successful colonization of gut	[30]
*L. reuteri *ATCC 55730	Healthy volunteers	10^8^	28-day trial; gut colonization; immune modulation	[31]
*B. animalis* subsp. *lactis* BB-12 *Streptococcus thermophilus *	Children with rotavirus diarrhea	10^8^	Reduction in incidence of acute diarrhea and rotavirus shedding	[32]
*B. lactis *strain BB12	Healthy breastfed infants	10^6^	Prophylactic against acute diarrhea	[33]
*B. anima*lis subsp. *lactis* BB-12 *L*. *reuteri* ATCC 55730	Children with acute diarrhea	10^7^	12-week trial; fewer and shorter episodes of diarrhea	[34]
*L. delbrueckii* subsp. *bulgaricus* *S. thermophilus *	Healthy volunteers	10^7^–10^9^ (*L. delbrueckii)* 10^8^–10^10^ (*S. thermophilus*)	12-day trial; effective gut colonization	[35]
*B. animalis *subsp. *lactis* BB-12 *L. paracase*i subsp. *paracasei* CRL-431	Healthy volunteers	10^8^–10^11^	7-week study; fecal recovery increases with increase in dose	[36]
*L. reuteri* DSM 17938 *L. rhamnosus * GG	Healthy volunteers	10^9^	3-week trial; increase in fecal recovery of viable lactobacilli	[37]
*Pediococcus acidilactici *MTCC 5101	Healthy human volunteers	10^8^–10^10^	4-week trial; colonization and fecal recovery increases with time	Present study
